# Phase I/II clinical trial of anti-OX40, radiation and cyclophosphamide in patients with prostate cancer: immunological analysis

**DOI:** 10.1186/2051-1426-1-S1-P255

**Published:** 2013-11-07

**Authors:** Magdalena Kovacsovics-Bankowski, Lana Chisholm, Jonna Vercellini, Marka Crittenden, Scot Lary, Brendan Curti, Andrew Weinberg

**Affiliations:** 1 Earle A. Chiles Research Institute, Providence Cancer Center, Portland Providence Medical Center, Portland, OR, USA

## Introduction

OX40, a member of the Tumor Necrosis Factor Receptor superfamily is a potent co-stimulatory molecule. OX40 engagement increases T cell proliferation, effector function and survival. Pre-clinical studies have shown that OX40 agonist synergizes with radiation and cyclophosphamide to increase survival. A phase I clinical trial has shown that anti-OX40 mAb was well tolerated and induced proliferation of CD8 and CD4 non-Treg cells in the peripheral blood (PB).

## Methods

This trial was design to evaluate the toxicity and the effect on peripheral blood lymphocytes of cyclophosphamide and radiation in combination with anti-OX40 agonist in patients with metastatic castrate- and chemotherapy-resistant prostate cancer. The immunological analysis was performed on peripheral blood lymphocytes (PBL) using a multi-color flow analysis panel containing CD3, CD4, CD8, CD95, CD25, CD38, HLA-DR and the intracellular markers, FoxP3 and Ki-67.

## Results

Anti-OX40, Cyclophosphamide and radiation had manageable safety and tolerability profile. Transient decreases in PSA were observed in 4/9 patients. Four patients had an increase in PSA DT. In 5/9 patients, bone and lymph node metastases were radiographically stable during the study observation. The immunological response measured in the PB shows a 2-3.5-fold increase in the proliferating CD4+ CD95+ T cells, mostly in the FoxP3- population. In the 3rd cohort, there was also a 3 fold increase, in CD4+ FoxP3+ T cells proliferation (Treg). The proportion of cycling CD8+ CD95+ T cells peaked with a 5-6-fold increase of cycling cells. NK cell proliferation was also observed, with a 2-4.5 fold proliferation increase. In the first anti-OX40 clinical trial, we have observed that the administration of anti-OX40 antibody increased the activation status of the CD8+ T cells as measured by the co-expression of CD38 and HLA-DR on the cycling cells. A similar trend was observed in this study. In some patients, the administration of anti-OX40 modified the cytokine profile of PBMC. We have observed an increase in IFNγ and IL-2, with stable TNFα secretion and a decrease in IL-6, IL-10 and IL-17.

## Summary

Administration of anti-OX40 with radiation and cyclophosphamide has not affected the degree of proliferation of PBL. We have observed a similar fold increase in the proliferation of CD4+ FoxP3-, CD8+ and NK cells as in the first clinical trial. With the exception of cohort #3 (highest dose of cyclophosphamide), there was no change in the proliferation of CD4+ FoxP3+ T cells (Treg). There was a trend towards a higher percentage of cycling CD8+ T cells expressing the activation markers CD38 and HLA-DR.

